# Primary angiosarcoma arising in the sinus of Valsalva: A case report

**DOI:** 10.1016/j.ijscr.2024.109308

**Published:** 2024-01-30

**Authors:** Kenichi Morimoto, Shigeto Miyasaka, Yosuke Ikeda, Rikuto Nii, Yoshikazu Fujiwara

**Affiliations:** Department of Cardiovascular Surgery, Tottori Prefectural Central Hospital, 730 Ezu, Tottori 680-0901, Japan

**Keywords:** Primary aortic tumor, Angiosarcoma, Sinus of Valsalva aneurysm

## Abstract

**Introduction:**

Primary angiosarcoma of the aorta, particularly within the sinus of Valsalva, is uncommon, with no documented instances of primary angiosarcoma. The absence of apparent clinical manifestations in this severe condition makes it challenging to diagnose, often resulting in a poor prognosis.

**Case presentation:**

A 60-year-old patient underwent procedures for fistula closure and coronary artery bypass grafting, which resulted in the rupture of an aneurysm within the sinus of Valsalva. Computed tomography examination 5 years after the procedure suggested no pathological abnormalities. Nevertheless, the patient required repeat surgery at 67 years due to the observed expansion of the sinus of Valsalva aneurysm noted during a clinical evaluation, prompted by elevated levels of inflammatory markers. Exploration of the residual aneurysmal locus within the sinus of Valsalva revealed an intraluminal thrombus devoid of any demonstrable hemodynamic access into the aneurysmal sac. Histopathological assessment of the aneurysmal wall confirmed a definitive diagnosis of primary angiosarcoma within the sinus of Valsalva. After surgery, the patient exhibited pyrexia. Magnetic resonance imaging substantiated multifocal osseous metastases, corroborated by histological analysis following a bone biopsy, confirming a diagnosis of angiosarcoma. Therefore, adjuvant chemotherapy with paclitaxel was initiated. After 1 year, a sustained state of disease stability was noted.

**Discussion:**

In this case, the need for surgical intervention, based on an expanded sinus of Valsalva aneurysm, culminated in the unanticipated detection of primary angiosarcoma.

**Conclusion:**

Neoplastic etiologies may plausibly underlie the pathogenesis of aneurysm formation in cases where the etiology remains obscure in the early stages of therapeutic intervention.

## Highlights

• Primary aortic angiosarcoma arising in the sinus of Valsalva is uncommon.
• It exacerbates the challenge of early diagnosis and worsens the prognostic outlook.
• Neoplastic etiologies may plausibly underlie the pathogenesis of aneurysm formation.

## 1 Introduction

Primary angiosarcoma of the aorta, especially in the sinus of Valsalva, is rare. However, primary angiosarcoma affecting the sinus of Valsalva has not been reported yet. The absence of clinical symptomatology complicates the prompt identification of this condition, exacerbating the challenge of early diagnosis and worsening the prognosis [[1], [2], [3]].

Herein, we report a case of primary angiosarcoma within the sinus of Valsalva. The work has been reported in line with the SCARE criteria [4].

## 2 Case presentation

**Fig. 1:**
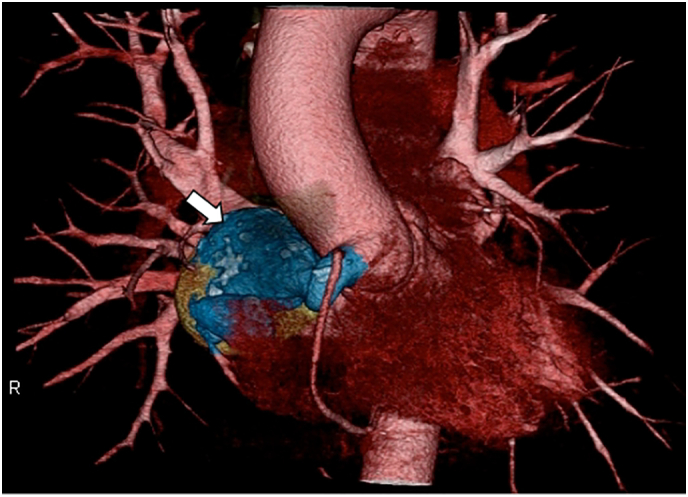
Preoperative computed tomography for the initial surgical procedure. An aneurysm measuring 53 × 48 mm is seen in proximity to the right aspect of the left atrium (white arrow), juxtaposed with the superior dorsal aspect of the right atrium and connected through a tubular structure originating from the sinus of Valsalva in the lower extremity of the ostium of the right coronary artery. Concomitantly, a fistulous communication is seen extending into the right atrium.

**Fig. 2:**
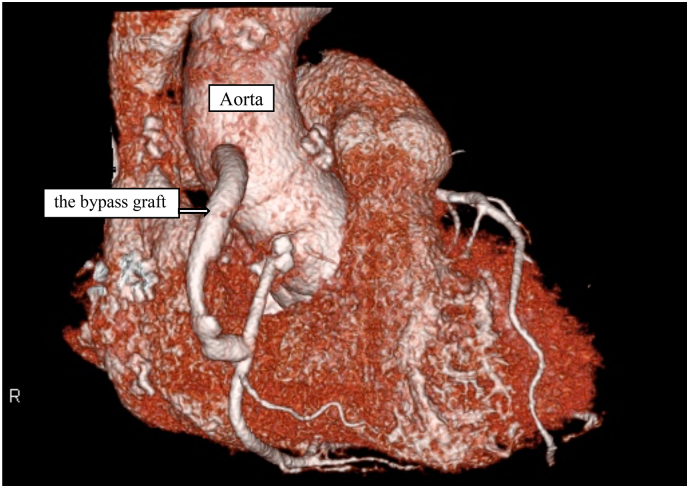
Postoperative computed tomography for the initial surgical procedure The aneurysm within the sinus of Valsalva appears thrombosed and devoid of any discernible contrast enhancement. Furthermore, the bypass graft originating from the ascending aorta and directed toward the second right coronary artery remains open and unobstructed.

A 67-year-old man presented with an unremarkable family history and underwent surgery at 60 years of age for a sinus of Valsalva aneurysm complicated by fistulous communication with the right atrium. Considering the invasiveness of complete aneurysm resection, a palliative surgical approach was used. This procedure included right coronary artery ostium closure, simultaneously repairing the initial segment of the sinus of Valsalva aneurysm. The ruptured orifice of the sinus of Valsalva aneurysm was concurrently addressed through meticulous patch formation, employing an artificial vessel (J-graft ®; Japan Lifeline, Tokyo, Japan). After aneurysmal decompression, the second branch of the right coronary artery was revascularized using a great saphenous vein graft (Fig. 1, Fig. 2).

During the initial surgery, the aneurysm was not incised; hence, its detailed characteristics remained unknown. Visual findings were consistent with those of a true aneurysm. Postsurgical surveillance was conducted over a quinquennial period via computed tomography (CT), during which no aberrant findings were documented.

**Fig. 3:**
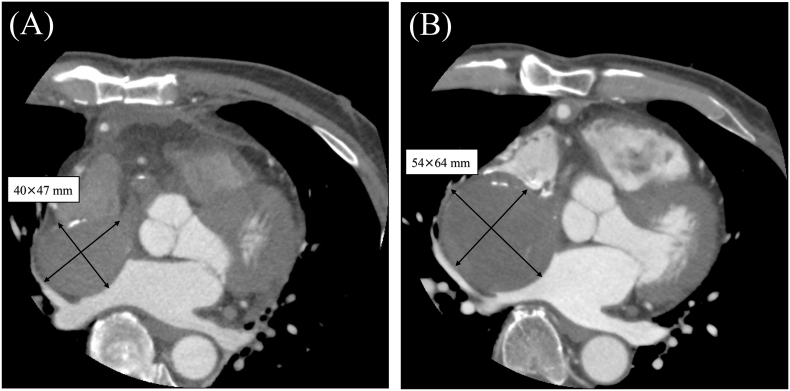
Contrast-enhanced chest computed tomography (CT). In the initial postoperative CT, the aneurysm had a diameter of 40 × 47 mm (A). However, a conspicuous aneurysm, measuring 54 × 64 mm in diameter (B), can be seen within the sinus of Valsalva and appears conspicuously devoid of any appreciable contrast enhancement. CT, computed tomography.

At 67 years, the patient was examined because of elevated inflammatory marker levels, and an enlarged sinus of Valsalva aneurysm was found. He was referred to our department for further examination and treatment. The patient was asymptomatic, and no other abnormalities were observed. Comprehensive hematological analysis revealed no significant abnormalities except for a C-reactive protein level of 18.68 mg/dL. Transthoracic echocardiography revealed a 64.8 × 57.5-mm-sized mass in the sinus of Valsalva, with no asynergy or valvular disease. Contrast-enhanced CT revealed an aneurysm in the sinus of Valsalva with a diameter of 54 × 64 mm without contrast effect; no abnormalities were observed in other areas, including the right atrium (Fig. 3). Cardiac catheterization revealed no contrast effect in the aneurysm in the sinus of Valsalva on aortography. The bypass graft remained open and unobstructed, with no inflow vessels into the aneurysmal cavity. No significant stenosis of the other coronary arteries was observed. Additionally, magnetic resonance imaging (MRI) and positron emission tomography (PET)-CT scans were not conducted preoperatively.

**Fig. 4:**
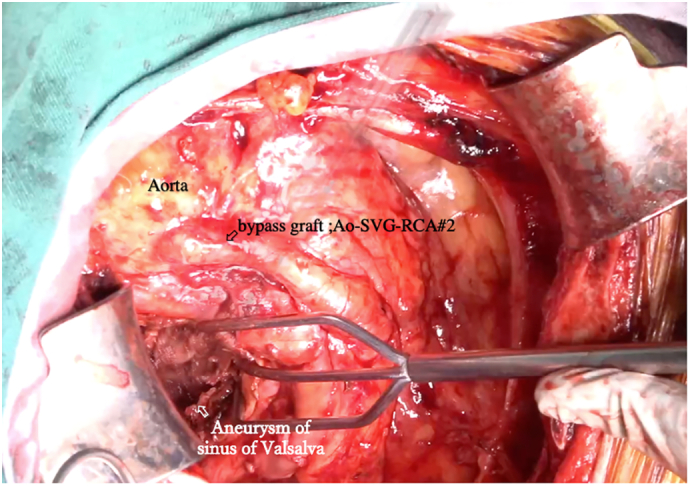
Surgical findings. The aneurysm, measuring approximately 6 cm in diameter, is situated on the right anterior facade of the right coronary artery ostium. The aneurysmal wall was excised, followed by meticulous evacuation of the intraluminal thrombus. Further examination of the aneurysmal cavity yielded no evidence of hemodynamic communication with the aorta, leading to the conclusion of a noncommunicating state.

Surgical intervention was required due to the risk of rupture from expansion at 20 mm over 2 years. Despite unclear exact causes, assessments from contrast-enhanced CT, echocardiography, and aortic angiography showed no inflow into the aneurysm. These assessments led us to consider factors such as microvascular flow from vasa vasorum and inflammatory or infectious origins rather than a reopening of the previously closed portion from the prior surgery. Median sternotomy revealed severe adhesions in the anterior aspect of the right atrium and ascending aorta. Cardiopulmonary bypass (CPB) was established. An approximately 6 cm diameter aneurysm on the right anterior facade of the right coronary artery ostium was identified. A longitudinal incision along the anterior surface of the sinus of Valsalva (about 4 cm) was made, parallel to the interatrial groove, followed by intraluminal thrombus evacuation. Subsequent examination of the aneurysmal cavity yielded no evidence of hemodynamic communication with the aorta, indicating a noncommunicating state. Histopathological and bacterial culture analyses were conducted on a segment of the mass wall. The residual aneurysm remained open, and the patient was gradually weaned off CPB (Fig. 4).

**Fig. 5:**
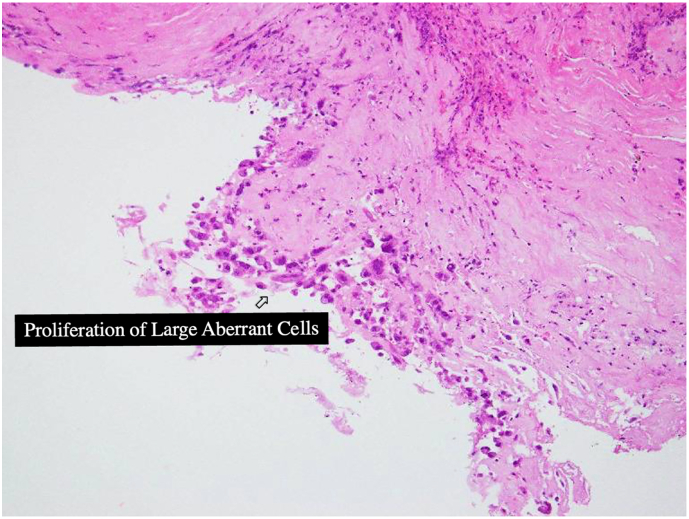
Pathological findings. Atypical cellular entities associated with the intimal surface of the aorta are discernible within the pathological domain. A subset of these atypical cells coalesced to form structures similar to the vascular lumen (hematoxylin and eosin stain, ×200).

Postoperative pathological analysis revealed the proliferation of substantial aberrant cells along the intimal surface of the aorta; some atypical cells coalesced to form structures resembling the vascular lumina. Immunostaining test results were positive for AE1/3, CD31, p53 (DO7), and Ki67 (MIB1), confirming angiosarcoma. The resection margins were negative; however, only a minimal portion of the aneurysm wall was available for pathological examination, limiting the significance of assessing the degree of infiltration at the tissue margins (Fig. 5).

The patient achieved favorable postoperative convalescence and was discharged 18 days following surgery. After discharge, the patient exhibited pyrexia. Subsequent MRI and PET-CT, performed to elucidate the etiology of the febrile state, revealed radiographic evidence of multifocal osseous metastatic involvement affecting the lower thoracic, lumbar, and sacral vertebrae. The patient has since undergone adjunctive chemotherapy with paclitaxel (100 mg/m^2^) with a stable clinical condition after 12 months.

## 3 Discussion

Primary aortic neoplasms are uncommon [[1], [2], [3]], with the abdominal aorta as the predominant location, accounting for 40 % of the cases [5]. In this case, the diagnosis of an enlarged sinus of Valsalva aneurysm was confirmed by postsurgical pathological examination. Although cases of cardiac tamponade stemming from fistula formation due to neoplastic growth or invasion—and ischemic symptoms caused by arterial stenosis or embolization—have been documented [6,7], early diagnosis remains challenging as most cases remain asymptomatic until substantial encroachment occurs in adjacent normal tissues. More than 50 % of the cases present with metastases during primary tumor diagnosis [8]; this was also observed in our case, wherein multiple bone metastases were postoperatively identified.

Although MRI, PET-CT, and transesophageal echocardiography are valuable diagnostic modalities for detecting neoplastic lesions [1,2,9], preoperative diagnosis remains elusive as these lesions frequently present as aneurysms or atherosclerotic occlusive lesions. In this case, contrast-enhanced CT and transthoracic echocardiography were performed preoperatively; however, a definitive preoperative diagnosis remained elusive due to the lack of suspicion of a tumorous lesion as the cause before the surgery. Angiosarcoma became apparent after the surgery and was identified incidentally due to the recurrence of the aneurysm. Furthermore, during the initial surgery, whether the cause of the sinus of Valsalva aneurysm was due to angiosarcoma remains unclear because the first surgery focused solely on closing the aneurysm and its fistula without removing it. However, considering the extremely poor prognosis associated with angiosarcoma, it is unlikely that angiosarcoma existed at the time of the initial surgery, as the patient's condition remained stable without disease progression for several years.

While primary aortic angiosarcoma lacks a standardized treatment regimen, the typical approach for localized tumors involves considering surgical excision, followed by adjuvant chemotherapy or radiotherapy as viable options. However, in this case, with no initial suspicion of a neoplastic lesion, achieving complete excision of the tumor was not possible. Subsequent postoperative PET-CT revealed accumulation in the remaining aneurysm wall, suggesting the possibility of tumor residue. Additionally, with the presence of distant metastasis, systemic chemotherapy was deemed more effective, as radiotherapy was not pursued. The choice of adjuvant therapy as part of a multidisciplinary protocol in the context of primary aortic angiosarcoma is a complex decision influenced by the rarity and aggressiveness of the disease. The optimal treatment strategy is not well-established due to the scarcity of aortic angiosarcoma cases. However, systemic chemotherapy, particularly using paclitaxel, has been explored in the management of angiosarcomas in various anatomical locations. The decision to use paclitaxel as a systemic chemotherapeutic agent is supported by its effectiveness in treating soft tissue sarcomas, including angiosarcomas. Paclitaxel shows anti-angiogenic properties, making it a plausible choice for targeting the highly vascular nature of angiosarcomas. Moreover, the use of paclitaxel in other sarcoma subtypes and its ability to disrupt microtubule function contribute to its consideration as a therapeutic option [[10], [11], [12]]. Therefore, postoperative treatment was initiated with weekly paclitaxel (100 mg/m^2^). The patient exhibited a stable disease state for 12 months following the initiation of postoperative chemotherapy.

Aortogenic angiosarcoma is a rare condition, and establishing a standardized management approach remains an arduous endeavor. According to a previous study, aneurysms of the sinus of Valsalva are relatively infrequent, accounting for 0.09 % of postmortem autopsies and 0.38–1.5 % of the cardiac surgery cases. The study also reported that while congenital anomalies represent the predominant etiological factor, secondary causes include trauma, infective endocarditis, syphilis, cystic necrosis of the tunica media, and arterial wall weakening due to arteriosclerosis [13]. The present case is the only reported instance of a sinus of Valsalva aneurysm with concomitant neoplastic progression. Nevertheless, in retrospect, the inflammatory marker levels were elevated, suggesting the possibility of a non-typical aneurysm. However, since the influence of malignant tumors was not considered, qualitative diagnostic tests such as MRI or PET-CT were not conducted as part of preoperative investigations. This case highlights the significance of including malignancy in the differential diagnosis for sinus of Valsalva aneurysms with elevated inflammatory markers. In the future, we aim to emphasize the need for qualitative examinations, such as MRI and PET-CT, in cases of sinus of Valsalva aneurysms with elevated inflammatory marker levels.

## 4 Conclusion

The lack of discernible clinical manifestations of primary aortic angiosarcoma arising in the sinus of Valsalva poses a challenge in promptly identifying this condition, accentuating the poor prognosis. In our case, the need for preoperative intervention—triggered by the expansion of a sinus of Valsalva aneurysm—culminated in the unanticipated diagnosis of primary angiosarcoma. This finding indicates that neoplastic pathogenesis may constitute a conceivable etiology in cases where the cause of aneurysms remains complicated, precluding a definitive preoperative diagnosis.

## Consent

Written informed consent was obtained from the patient for publication and any accompanying images. A copy of the written consent is available for review by the Editor-in-Chief of this journal on request.

## Ethical approval

This study is exempt from ethical approval in our institution.

## Funding

This research did not receive any specific grant from funding agencies in the public, commercial, or not-for-profit sectors.

## Author contribution

All authors participated in the patient's care, performed the surgeries, wrote the draft, revised the manuscript, and read and approved the final manuscript.

## Guarantor

Kenichi Morimoto.

## Research registration number

Not applicable.

## Conflict of interest statement

None.
